# Hiatus Hernia as a Complication of Gastric Banding: A Systematic Review and Meta-Analysis

**DOI:** 10.7759/cureus.29704

**Published:** 2022-09-28

**Authors:** Yacoub Majdoubeh, Falah Abu Hassan, Minas Abu Alhalawa, Saja Aljobouri

**Affiliations:** 1 Emergency Department, Kingston Hospital NHS Foundation Trust, London, GBR; 2 Internal Medicine, Texas Tech University Health Sciences Center, Amarillo, USA; 3 Neuroscience, Queen Mary University of London, London, GBR; 4 Internal Medicine, University of Jordan, Amman, JOR

**Keywords:** banding, gastric, laparoscopic, hernia, hiatus

## Abstract

Worsening hiatus hernia (HH) symptoms have been well recognized as a complication of gastric banding, however, it has not yet been explored whether gastric banding plays a role in the development of HH de novo in patients undergoing gastric banding. From the 696 studies identified, five studies met the eligibility criteria and were included. Data was extracted from PubMed, Embase, Medline, HMIC, and Web of Science databases. The pooled complication rate was evaluated along with 95% confidence intervals (95% CIs). The meta-analysis was performed using the Cochrane RevMan tool (Cochrane, London, UK). Heterogeneity was tested using the I^2^ index for each outcome. All the included studies assessed HH incidence among followed-up patients who needed a re-operation for upper gastrointestinal symptoms. Between-study variability was high (I^2^ = 94%, Chi^2^ = 68.92, df = 4, < 0.00001, Tau2=1.91). Complication rate ranged between 0.24% to 5.55%; pooled complication rate was 2.17% CI 95% (0.90 - 3.44%) P = 0.0008. The included studies show a comparable rate of post-operative HH; the fact that HHs can become symptomatic following the adjustable gastric banding (AGB) procedure indicates that AGB plays a role in creating symptomatic hiatal hernias at the very least. Further research is needed to underpin the mechanism and confirm causation. However, this complication should potentially be discussed with patients opting for this kind of operation as it can be a reason for re-operation.

## Introduction and background

Obesity is a serious medical condition of having a body mass index of 30 or higher, and it has been rising rapidly among adults and children in the last 10 years. It is linked to chronic co-morbidities and higher chances of early mortality [1], which warrants immediate interventions in some cases, some of which can be surgical and referred to as bariatric or metabolic surgeries [2]. The eligibility criteria for bariatric surgeries were determined by the National Institutes of Health to avoid unnecessary surgeries [3], and only 1% of those eligible undergo surgery, potentially due to the fear of encountering surgical complications [1].

Bariatric surgeries are either malabsorptive or calorie restrictive. Malabsorptive surgeries aim to decrease the absorption of the food consumed. Calorie restrictive surgeries decrease the size of the stomach and restrict calorie intake, such as adjustable gastric banding (AGB) [3]. AGB surgery is a minimally invasive surgery where the upper part of the stomach is held by a restrictive band forming a pouch that is separated from the rest of the stomach by the band. Compared to other bariatric surgeries, laparoscopic adjustable gastric banding (LAGB) was considered a safer and less complicated surgery than some of the other bariatric surgeries such as Roux-en-Y gastric bypass and biliopancreatic diversion procedure; this is believed to be the reason why it has been increasingly performed worldwide [4]. AGB alongside Roux-en-Y gastric bypass and sleeve gastrectomy are the most popular and commonly performed bariatric procedures [5].

Although LAGB is a minimally invasive procedure compared to other bariatric surgeries [4], revision surgery rates due to postoperative complications, such as band slippage, pouch dilation, and band erosion among others, are considered relatively high [6]. Revision surgeries may include band replacement or conversion to other bariatric procedures [7]. LAGB has a high rate of re-operation (up to 32%) with the most common reason being band slippage/gastric prolapse [8]. Furthermore, several studies have reported complication rates ranging from 1% to 26% with the most common of these being band slippage and pouch dilatation [9], and in turn, it was reported that 27% of prolapses and 53% of pouch dilatations are associated with hiatal hernias [9].

Hiatus hernia (HH) is a condition where abdominal organs, such as the stomach bulge into the middle compartment of the chest through the esophageal hiatus [10]. HH development is a complication that has been classically associated with bariatric procedures other than gastric banding. However, there is a growing body of evidence that suggests that hiatal hernia development might be a direct complication of gastric banding.

The purpose of this study is to systematically assess the evidence with regard to hiatal hernia as a direct complication of gastric banding.

## Review

Methods

Data Collection

The data were extracted from PubMed, Embase, Medline, HMIC, and Web of Science databases using search keywords such as bariatric, banding, and diaphragmatic or hiatal hernia.

No limits were imposed on the time, language, and publication status during the search. All the references in the included studies were hand-searched for relevant articles. Medical Subject Headings (MeSH) terms were used with keywords for gastric banding and hiatal hernia. We included cross-sectional studies and observational studies. Only studies where patients had the initial banding performed and then followed up for complications were included in the meta-analysis, without eliminating patients who had not had their hiatus inspected in the primary operation. All studies which assessed symptomatic patients only were excluded.

**Figure 1:**
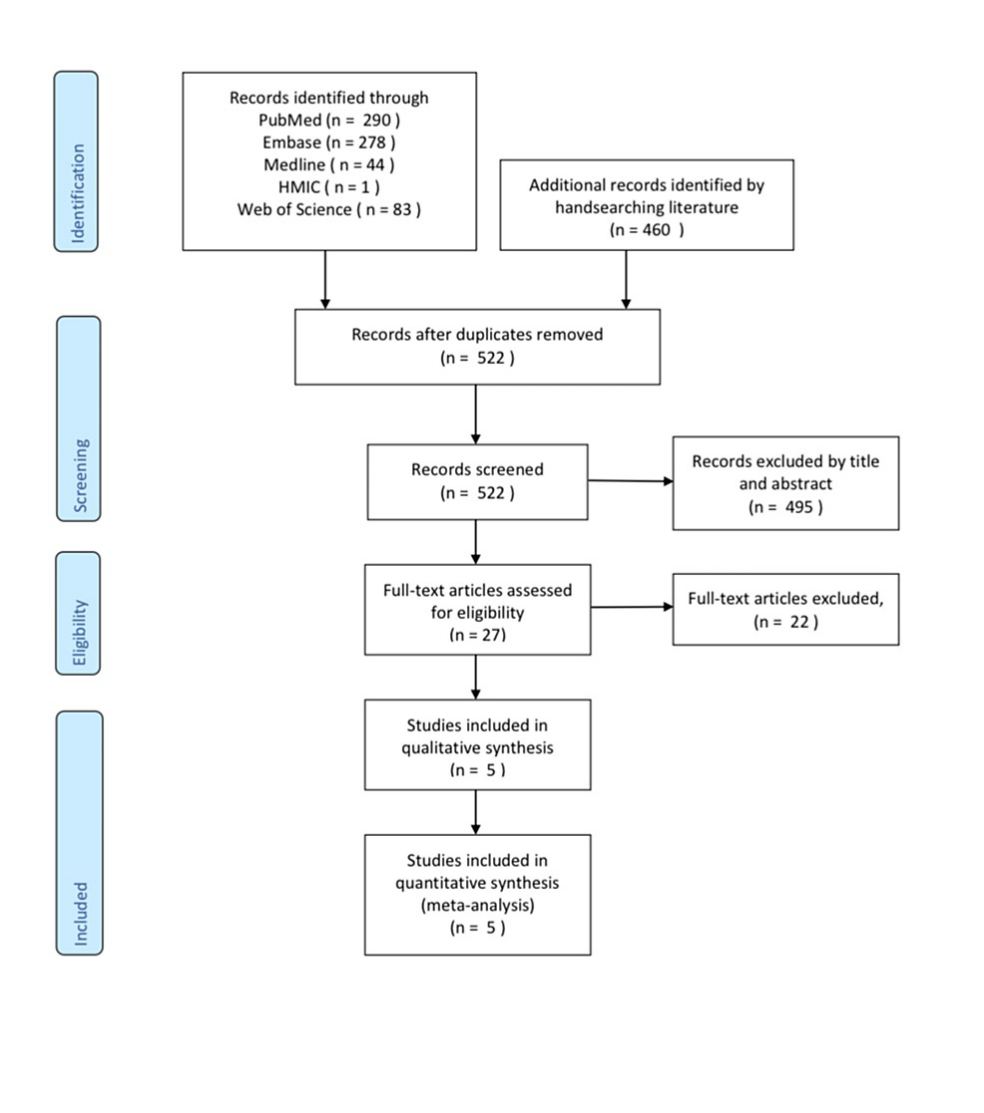
PRISMA Flowchart for the Identification, Screening, Eligibility and Inclusion of Studies PRISMA: Preferred Reporting Items for Systematic Reviews and Meta-Analyses

Figure 1 shows the Preferred Reporting Items for Systematic Reviews and Meta-Analyses (PRISMA) chart of the screened studies; 696 potentially eligible studies were identified. After excluding duplicates, 522 titles and abstracts were evaluated by two reviewers independently after which 27 publications were retrieved, and their manuscripts were thoroughly evaluated for inclusion. A total of five studies met all eligibility criteria and were included in this meta-analysis by agreement. The demographics of the patients included in each of those studies are shown in (Table 1).

**Table 1:** Summary of Studies’ Findings 1. In this study, the age of participants was reported as a median and a range. 2. This study reported the age of participants as a mean and a range. The standard deviation was approximated, using the range provided, via the equation present in section 7.7.3.6 of “Cochrane Handbook for Systematic Reviews of Interventions”. HH: hiatus hernia; LAGB: laparoscopic adjustable gastric banding; SD: standard deviation.

Study	Number of Participants	Number of Hiatal hernias (HH)	Inspection for HH in primary LAGB (pLAGB)	Mode of detection of HH	Male (%)	Mean Age (+/- SD)
Brown et al. [8]	425	1	Yes	Barium Swallow	19	^1 ^43 ± 13.25
Gulkarov et al. [9]	1298	72	Yes	Esophagography and upper endoscopy	32.4	40.6 ± 12.6
Azagury et al. [11]	685	12	Yes	Standard upper gastrointestinal contrast study (UGI)	Not Reported	Not Reported
Parikh et al. [12]	749	17	No	Esophagography	29.1	^2 ^42.3 ± 13.5
Beitner et al. [13]	3876	60	No	Esophagography	14.4	43.22 ± 11.76

Data Analysis

Two independent reviewers extracted the data from the included studies and the conflict was resolved by agreement. Data included first author, year of publishing, methods, participants, and total number of occurrences of post-operative hiatal hernia.

The meta-analysis was performed using the Cochrane RevMan tool (Cochrane, London, UK). Pooled complication rate was evaluated along with 95% confidence intervals (95% CIs). All parameters were summarized using the random-effects model. Heterogeneity was tested using the I2 index for each outcome.

Results 

**Figure 2:**
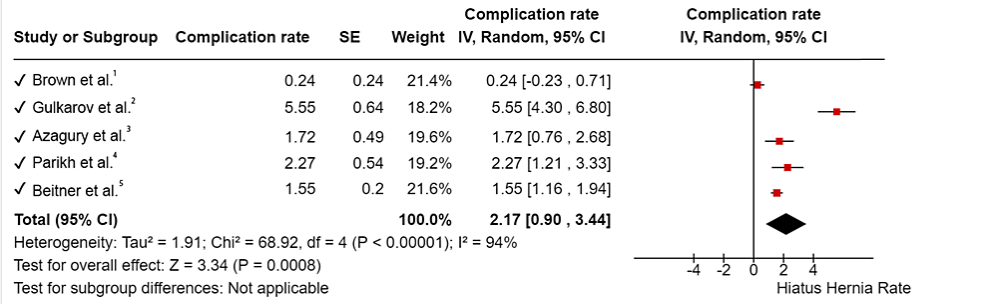
Meta-analysis of the Rate of Hiatus Hernia Following Gastric Banding [8,9,11-13]

Due to the expected variation between studies, random-effects meta-analyses were carried out using the total sample size and number of positives. The meta-analysis indicated that between-study variability was high (I2 = 94%, Chi2 = 68.92, df = 4, < 0.00001, Tau2=1.91). Complication rate ranged between 0.24% to 5.55%; pooled complication rate was 2.17% CI 95% (0.90 - 3.44%) P = 0.0008. These results are summarized in the forest plot in (Figure 2).

Discussion

This is the first systematic review and meta-analysis of this subject. We included five studies that we assessed to be combinable. The main and only end-point we were looking for was the rate of HH occurrences following the placement of an adjustable gastric band. In this study, we have concluded that gastric banding is associated with the formation of HH postoperatively.

Since its description in 1994 by Belachew et al., AGB has become one of the main bariatric procedures to be performed [14]. The reversibility of the technique, in addition to its efficacy and safety, makes it an appealing option for many. That being said, it is not without its complications, and a growing body of evidence is demonstrating that AGB is related to complications, such as HH development, which is a complication that is typically associated with other bariatric procedures. The mechanisms behind LAGB complications, such as HH, are still being investigated [11]. Several studies have proposed reasons behind the development of HH after gastric banding. Brown et al. hypothesized that the main issue is excessive high pressure due to eating too large of a volume or too quickly, within the proximal pouch; such forces could cause distention of the proximal pouch which could result in it pushing up on the pharyngoesophageal ligament, causing hiatal laxity or a secondary sliding HH [8].

Gulkarov et al. proposed that crural defects at the esophageal hiatus could allow for the periodic displacement of the gastroesophageal junction up into the thorax [9]. Azagury et al. proposed that complications such as concentric pouch dilation, esophageal dilation, and HH are likely to have the same etiology and classified them together as “backpressure syndrome”; depending on the weakest link, the pressure will induce concentric pouch dilation, esophageal dilation, or a combination of three [11]. The presence of a new HH after LAGB and the role of LAGB in potentially creating hiatal hernias has not been thoroughly assessed in the literature; however, Azagury et al. identified 12 patients out of 695 who needed to undergo reoperation for HH, and who had no evidence of hiatal hernia radiographically or by visualization during the primary operation [11].

Parikh et al. reported gastric prolapse being the most common delayed complication after LAGB, occurring in 2.9% of patients; 27% of these patients had a concurrent hiatal hernia that necessitated repair at re-operation [12]. Furthermore, it was reported that pouch dilatation occurred in 2% of the patients, and this was associated with a concurrent HH in 53% of the cases [12]. It was, however, stipulated that HH might have been under-diagnosed at the primary operation, as it was not consciously searched for [12]. Beitner et al. reported that HH is the second most common reason for re-operation after LAGB, sometimes leading to two or three reoperations [15]. In another study, where a HH was repaired when detected during the first operation, it was also reported that HH is the second most common indication for revisional surgery [13]. This does raise the suggestion that even if the presence of a HH preceded the placement of the adjustable gastric band, it could be responsible for the conversion of the hernia into a symptomatic one at the very least.

Gulkarov et al. compared the re-operation rate for HH, with or without pouch dilatation or slip, in two groups of patients. Those who underwent LAGB and HH repair at the first operation and those who underwent only LAGB at the first operation. Although the re-operation rate was considerably higher for the LAGB-only group (5.6%), it was still reported that 1.7% of patients needed re-operation for HH development in the LAGB and HH repair group [9].

Despite being excluded from the meta-analysis data, the following studies do also offer some insight on the subject, and the findings are included in the text and are as follows: Cruiziat et al. assessed 22 patients experiencing upper gastrointestinal symptoms after LAGB by high-resolution manometry; a manometric HH was detected in 14 of these patients [16]. In another analysis of 143 patients presenting with upper gastrointestinal symptoms or an unsatisfactory outcome after LAGB, an incidence of 2% of hiatal hernias using stress barium contrast swallow was reported [17]. Another study retrospectively reviewed the first 40 consecutive patients out of 1275 patients who underwent LAGB and who developed certain complications. It was found that 64% of patients with pouch dilatation had a concurrent HH [18]. It was, however, also stipulated that hiatal hernias might be under-diagnosed at the time of the primary operation [18]. Interestingly in one study, a higher rate of HH being present at revisional LAGB than at primary LAGB was reported (26.3% vs 18.9%) [19].

Limitations

Only three of the included studies actively checked for a preoperative HH. Of the studies in which the presence of a HH was not checked preoperatively or intra-operatively, the emergence of a postoperative HH would have been attributed to an otherwise precedent undetected asymptomatic hernia. Nevertheless, the close rate of post-operative HH in all the included studies, and the fact that HHs can form or become symptomatic following the AGB placement procedure, indicate that AGB plays a role in creating HH at the very least. All the included studies assessed HH incidence among followed-up patients who needed a re-operation for upper gastrointestinal symptoms. Although this can reflect the rate of symptomatic HH, it does miss many more patients who might have developed an asymptomatic HH.

## Conclusions

Although HH development has been classically established as a direct complication of certain bariatric procedures, it was not classically associated with LAGB. A growing body of evidence suggests that symptomatic HH development could be a direct complication of LAGB, as evidenced by the studies in this meta-analysis. Although further research is needed to underpin the mechanism of symptomatic HH development following LAGB and to confirm causation, symptomatic HH development as a complication of LAGB should be potentially discussed with patients opting for this kind of operation, especially since it may be a reason for re-operation.

## References

[1] Ma IT, Madura JA II (2015). Gastrointestinal complications after bariatric surgery. Gastroenterol Hepatol (N Y).

[2] Faria GR (2017). A brief history of bariatric surgery. Porto Biomed J.

[3] Medical Advisory Secretariat (2005). Bariatric surgery: an evidence-based analysis. Ont Health Technol Assess Ser.

[4] Galvani C, Gorodner M, Moser F, Baptista M, Chretien C, Berger R, Horgan S (2006). Laparoscopic adjustable gastric band versus laparoscopic Roux-en-Y gastric bypass: ends justify the means?. Surg Endosc.

[5] Mulita F, Lampropoulos C, Kehagias D (2021). Long-term nutritional deficiencies following sleeve gastrectomy: a 6-year single-centre retrospective study. Prz Menopauzalny.

[6] Kodner C, Hartman DR (2014). Complications of adjustable gastric banding surgery for obesity. Am Fam Physician.

[7] Ngiam KY, Khoo VY, Kong L, Cheng AK (2016). Laparoscopic adjustable gastric banding revisions in Singapore: a 10-year experience. Obes Surg.

[8] Brown WA, Burton PR, Anderson M, Korin A, Dixon JB, Hebbard G, O'Brien PE (2008). Symmetrical pouch dilatation after laparoscopic adjustable gastric banding: incidence and management. Obes Surg.

[9] Gulkarov I, Wetterau M, Ren CJ, Fielding GA (2008). Hiatal hernia repair at the initial laparoscopic adjustable gastric band operation reduces the need for reoperation. Surg Endosc.

[10] Mittal RK (1997). Hiatal hernia myth or reality?. Am J Med.

[11] Azagury DE, Varban O, Tavakkolizadeh A, Robinson MK, Vernon AH, Lautz DB (2013). Does laparoscopic gastric banding create hiatal hernias?. Surg Obes Relat Dis.

[12] Parikh MS, Fielding GA, Ren CJ (2005). U.S. experience with 749 laparoscopic adjustable gastric bands: intermediate outcomes. Surg Endosc.

[13] Beitner MM, Ren-Fielding CJ, Kurian MS (2014). Sustained weight loss after gastric banding revision for pouch-related problems. Ann Surg.

[14] Belachew M, Legrand MJ, Defechereux TH, Burtheret MP, Jacquet N (1994). Laparoscopic adjustable silicone gastric banding in the treatment of morbid obesity. a preliminary report. Surg Endosc.

[15] Beitner MM, Ren-Fielding CJ, Fielding GA (2016). Reducing complications with improving gastric band design. Surg Obes Relat Dis.

[16] Cruiziat C, Roman S, Robert M (2011). High resolution esophageal manometry evaluation in symptomatic patients after gastric banding for morbid obesity. Dig Liver Dis.

[17] Burton PR, Brown WA, Laurie C, Hebbard G, O'Brien PE (2010). Predicting outcomes of intermediate term complications and revisional surgery following laparoscopic adjustable gastric banding: utility of the CORE classification and Melbourne motility criteria. Obes Surg.

[18] Ponce J, Fromm R, Paynter S (2006). Outcomes after laparoscopic adjustable gastric band repositioning for slippage or pouch dilation. Surg Obes Relat Dis.

[19] Reynoso JF, Goede MR, Tiwari MM, Tsang AW, Oleynikov D, McBride CL (2011). Primary and revisional laparoscopic adjustable gastric band placement in patients with hiatal hernia. Surg Obes Relat Dis.

